# Safety assessment of omeprazole use: a review

**DOI:** 10.1590/1516-3180.2018.0019220318

**Published:** 2018-12-13

**Authors:** Marcela Forgerini, Stephania Mieli, Patrícia de Carvalho Mastroianni

**Affiliations:** I BSc. Pharmacist and Master’s Student in the Postgraduate Program on Pharmaceutical Sciences, Universidade Estadual Paulista (UNESP), Araraquara (SP), Brazil.; II Undergraduate Pharmacy Student, Universidade Estadual Paulista (UNESP), Araraquara (SP), Brazil.; III PhD. Pharmacist and Adjunct Professor, Department of Drugs and Medicines, Universidade Estadual Paulista (UNESP), Araraquara (SP), Brazil.

**Keywords:** Review, Drug-related side effects and adverse reactions, Proton pump inhibitors, Drug interactions, Treatment outcome

## Abstract

**BACKGROUND:**

Risks regarding hospital admission due to adverse drug reactions and drug interactions from use of omeprazole have been reported. The question guiding the present review was “Which adverse events occur in patients using omeprazole in a Food and Drug Administration-approved and/or off-label manner?” It was also proposed to evaluate the safety of use of omeprazole.

**DESIGN AND SETTING:**

Qualitative narrative review with critical evaluation, in a public university.

**METHODS:**

The PubMed, SCOPUS, LILACS, SciELO, EMBASE and EBSCO databases were searched on July 31, 2018. Studies evaluating adverse events were screened.

**RESULTS:**

72 articles were included, among which 58 reported on adverse drug events (47, adverse drug reactions; 5, drug interactions; and 6, situations of ineffectiveness). 28 adverse drug reactions not described in compendia and drug leaflets were described in these studies: myocardial infarction (6); stroke (2); spontaneous abortion (1); proliferative changes (1); chills (1); heart failure (1); thrombosis (2); and dementia (1), among others. Severe adverse reactions, for instance cardiac problems, Steven-Johnson syndrome and proliferative changes, were identified. The antiplatelet effects of drugs such as clopidogrel, in patients who underwent heart-related surgery, increased the risk of developing cardiac problems, such as cardiovascular death, myocardial infarction and stroke. In newly transplanted patients, decreased absorption of mycophenolate mofetil occurred, thus leading to rejection of transplanted organs.

**CONCLUSION:**

Use of omeprazole should be monitored primarily in patients with heart disorders using antiplatelet agents concomitantly, and in newly transplanted patients using mycophenolic acid, in order to avoid serious adverse reactions.

## INTRODUCTION

Proton-pump inhibitors (PPIs) such as omeprazole are one of the most widely prescribed classes of drugs worldwide. PPIs are indicated for treatment of ulcers with or without *Helicobacter pylori* infection; for treatment of gastroesophageal reflux, Zollinger-Ellison disease, dyspepsia, esophagitis and gastritis; and for prevention of peptic ulcers in patients receiving nonsteroidal inflammatory agents (NSAIDs) and in patients with upper gastrointestinal bleeding.^1^ Therefore, they are medications that are ever-present in gastroenterologists’ practice.^2^

Omeprazole is effective and safe most of the time.^1^ However, Mastroianni et al.^3^ found that omeprazole was the drug most commonly associated with hospital admission, in a survey on the prevalence of hospitalizations due to adverse drug reactions. In addition, the safety of a drug may change over time through increased use and according to patients’ characteristics. Therefore, risk assessment is required.^4^

This context can be elucidated from reports on abusive use of omeprazole and irrational prescription of this drug.^4^ Thus, there have been studies reporting on the risks (adverse events) of use of omeprazole, such as: (a) gastric proliferative changes;^5^ (b) increased creatinine and urea levels, leading to acute interstitial nephritis^6^^,^^7^^,^^8^ and increased risk of developing chronic kidney disease;^9^ (c) increased risk of asthma concomitant with gastroesophageal reflux;^10^ (d) increased risk of infection by *Clostridium difficile*;^11^^,^^12^^,^^13^ (e) decreased absorption of vitamin B;^14^ (f) steatorrhea caused by cystic fibrosis;^15^ (g) fracture with decreased calcium absorption;^16^^,^^17^ (h) gynecomastia;^18^ (i) hypomagnesemia;^19^ (j) hyponatremia;^20^ (k) spontaneous bacterial peritonitis;^21^ l) pneumonia;^22^ (m) anaphylactic reactions to omeprazole;^23^ and (n) risk of celiac disease.^24^

In addition, studies that evaluated the prevalence of hospital admission due to adverse drug events have cited omeprazole among the drugs that were possibly related to hospitalization, thus also suggesting that off-label use of omeprazole occurs frequently.^23^^,^^24^ Off-label use of drugs consists of their use for unapproved indications and usually occurs among polymedicated patients and as prophylactic gastric protection for use of some drugs, such as antimicrobials and nonsteroidal anti-inflammatory drugs.^25^^,^^26^^,^^27^ These off-label indications are for long-term use and are widespread and commonly prescribed in some countries,^28^ such as Brazil.

## OBJECTIVE

The purpose of this review was to evaluate the adverse outcomes relating to omeprazole use in clinical practice.

## METHODS

### Study design

We conducted a qualitative narrative review with critical evaluation, to answer the following guiding question: “Which adverse events occur in patients using omeprazole in a Food and Drug Administration (FDA)-approved and/or off-label manner?” Thus, we aimed to gather, organize and critically review articles on these topics, to include the highest level of scientific evidence.

### 1. Search of the literature and inclusion criteria

The search for studies was performed using the MEDLINE (via PubMed), LILACS, EMBASE (via Ovid), SciELO and SCOPUS databases and was conducted on July 31, 2018. During the search and selection process, there was no limitation on the time when articles were published. The languages were restricted to Portuguese, English and Spanish.

The following search strategies were used: (“Omeprazole” OR “Proton Pump Inhibitors”) AND (“Adverse Drug Reaction Reporting Systems” OR “Pharmacovigilance” OR “Drug-Related Side Effects and Adverse Reactions” OR “Risk Assessment” OR “Treatment Outcome” OR “Off-Label Use”). All descriptors used in these search strategies are Medical Subject Headings (MeSH terms). We included randomized clinical trials, phases I and II clinical trials, case-control studies, cohort studies, cross-sectional and quasi-experimental studies (clinical trials in which there was no comparator group for the intervention) evaluating adverse events from therapeutic or prophylactic use of omeprazole among individuals in all age groups whose health status was well defined and who were using omeprazole in an FDA-approved and/or off-label manner.

We excluded review articles, dissertations and theses, case reports, abstracts published in annals of events, editorials, letters to the editor, news and comments.

### 2. Selection process and data extraction

#### Types of participant

The target population comprised patients of any kind whose health status was well defined and who were using omeprazole in an FDA-approved and/or off-label manner. There was no age limitation.

#### Types of intervention

The interventions considered comprised use of omeprazole from the outset of treatment to clinical outcome, without restrictions on doses, therapeutic regimens or duration of use. In addition, it was proposed to include both preventive use and therapeutic use.

#### Types of outcome

The outcomes considered comprised any safety-related outcome, including adverse events, withdrawal due to adverse events, mortality and therapeutic ineffectiveness, i.e. adverse events in which the medicine used did not present any therapeutic response or its therapeutic response was lower than expected. Safety-related outcomes of all causes and omeprazole-related causes were considered.

After selecting potential articles in the databases, the titles and abstracts were reviewed by verifying patient exposure to omeprazole. The following variables were defined during the screening of articles: indication of use; study design; patient’s clinical condition; clinical outcomes, including all types of adverse events relating to use of omeprazole; recommendations; author; and year of publication.

The severity of adverse events was classified as described by the World Health Organization. In this definition, severe adverse reactions are harmful effects that occur during drug treatment and which can result in death, be life-threatening or lead to persistent or significant disability, congenital anomaly, clinically important effects, hospitalization or prolongation of hospitalization. Non-serious adverse reactions also fall within the concept of severe adverse reactions.^29^

The search for studies, selection of studies and extraction of data were performed by three authors, in triplicate independently, to avoid the presence of bias in the selection and exclusion of articles. In addition, the kappa function was applied to analyze the agreement rate.

### 3. Risk of bias assessment

For randomized clinical trials, risk of bias was evaluated using the Cochrane collaboration tool (RoB 1),^30^ which is based on seven domains: random sequence generation, concealment of allocation, blinding of participants and professionals, blinding of outcome assessors, outcome completeness, selective reporting of outcomes and other sources of bias. Each domain is judged as presenting low risk of bias, uncertain risk of bias or high risk of bias.

For case-control and cohort studies, we used the Newcastle-Ottawa tool. This provides evaluations in three domains: selection, comparability and outcome for cohort studies; and selection, comparability and exposure for case-control studies. Each item that is identified as presenting low risk of bias is given a “‘star”. There is a maximum of one “star” for each item within the “selection” and “exposure/outcome” categories; and a maximum of two “stars” for “comparability”. Therefore, each study can be classified with a maximum of nine “stars”, which corresponds to a low risk of bias.^31^

The cross-sectional and quasi-experimental studies included in this review were not evaluated, since there are no validated tools for analysis on these study designs.

## RESULTS

A total of 5,500 potentially relevant studies were identified. After reading the titles and/or abstracts, 4,746 studies were excluded because they did not meet the inclusion criteria. Another 218 were duplicates, and thus 536 studies were examined further.

**Figure 1.**
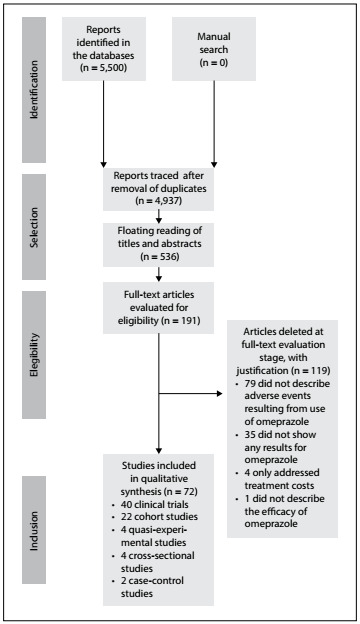
Flowchart of the stages of selection, skim-reading and full reading of the eligible articles.

It was not possible to access 2 of these 546 studies, because one of them is no longer indexed in the database and the other does not provide for the option to purchase and access the article. Our attempts to contact the authors of these two studies were unsuccessful. After screening the remaining articles, 191 studies were found to be eligible for complete text reading. After reading in full, 119 were excluded because they did not meet the inclusion criteria. Thus, 72 articles were considered eligible for the safety assessment on use of omeprazole, since they included all the variables that were being analyzed (Figure 1).

The proportion of overall agreement (kappa) observed in relation to making final decisions (inclusion and exclusion) from the database that included the screened articles was 0.807 (confidence interval, CI: 0.658-0.957).

Among these 72 studies, 58 reported on adverse drug events (ADEs): 47 studies on adverse drug reactions (ADRs), 5 studies on drug interactions (DIs) and 6 studies on therapeutic ineffectiveness (Table 1). The duration of use of omeprazole ranged from 5 days to 11 years in these studies. Only one study evaluated the off-label use of omeprazole.^41^

**Table 1.** Frequencies of adverse events resulting from indication of omeprazole that were reported in studies published up to 2016

Assessment	Type of ADE	Frequency	Description
Safety	ADR (n = 47)	Common reaction (≥ 1% and < 10%)	Headache, constipation, diarrhea, abdominal pain, back pain, flatulence, respiratory tract infection and maculopapular rash.^32^^,^^33^^,^^34^^,^^35^^,^^36^^,^^37^
		Uncommon reaction (≥ 0.1% and < 1%)	Eczematous eruption, insomnia, somnolence, urticaria, urticaria vasculitis and vertigo.^38^
		Rare reaction (≥ 0.01% and < 0.1%)	Angioedema, arthralgia, muscle pain, erythema multiforme, weakness, metallic taste in the mouth, allergic reaction, Steven-Johnson’s syndrome and thirst.^38^
		Post-marketing experience	Unstable angina, increased risk of fractures, cancer, cystitis, ulcerative colitis, stomatitis, abnormal renal function, hypergastrinemia, decreased levels of vitamin B12, increased creatinine levels, hypomagnesemia.^14^^,^^39^^,^^40^^,^^41^^,^^42^^,^^43^
		Potential events not described in omeprazole monograph (n = 28)	Miscarriage, proliferative changes, increased levels of chromogranin A, increased levels of fibroblast growth factor 2, chills, cardiovascular events (myocardial infarction, heart failure, stroke, ischemic stroke, pulmonary embolism and thrombosis), scarlet fever, hyperglycemia, mononucleosis infection, gastrointestinal bleeding, nasopharyngitis, otitis media, loss of libido, rhinitis, dementia, metabolic syndrome and hepatic steatosis, low sperm motility, increased risk of fibrosis progression, cirrhosis, hepatic decompensation and development of hepatocellular carcinoma.^44^^,^^45^^,^^46^^,^^47^^,^^48^^,^^60^^,^^65^^,^^66^^,^^67^
	DI (n = 6)	Omeprazole and clopidogrel: cardiovascular death, myocardial infarction, inhibition of the effect of clopidogrel, increased leukocyte and platelet levels and increased brain adverse events.^75^^,^^76^	
		Omeprazole and acenocoumarol: increased anticoagulant effect of acenocoumarol.^77^	
		Omeprazole and mycophenolate mofetil: reduced absorption of mycophenolic acid.^49^	
Efficacy	TI (n = 5)	Some patients did not respond to omeprazole therapy and continued with colitis symptoms and gastrointestinal discomforts. Omeprazole failed to control the gastric acidity of some patients.^13^	

ADE = adverse drug event; ADR = adverse drug reaction; DI = drug interaction; TI = therapeutic ineffectiveness. The frequency of adverse reactions was classified according to the leaflet of the reference drug product, except for the 28 studies for which there was no information on the leaflet.

A relationship was observed between use of omeprazole and increased risk of severe adverse events, such as development of coronary disorders that might lead to death. ^32^^,^^39^^,^^43^^,^^62^^,^^88^^,^^94^^,^^96^^,^^101^

Regarding the clinical outcomes of the studies, the safety (ADRs and DIs) and the therapeutic ineffectiveness can be correlated. Among the 62 studies included, 39 studies reported on ADRs, with 28 potential events that were identified during the post-marketing phase in relation to omeprazole (spontaneous abortion, proliferative changes and chills, among others); 6 studies demonstrated the drug interactions between omeprazole and clopidogrel or mycophenolate mofetil; and 5 studies described the therapeutic ineffectiveness that occurred with omeprazole (Table 2).

**Table 2.** Adverse events from approved use of omeprazole that were reported in the studies analyzed, published from 1994 to July 2018

	Adverse events	Participants (n)	Author, year
Clinical trials (28)	ADR: Diarrhea, vomiting and circulatory problems	Patients with reflux esophagitis (193)	Bate et al., 1995^50^
	ADR: Dyspepsia, flatulence, abdominal pain and diarrhea	Patients with active duodenal ulcer (180)	Marzio et al., 1995^51^
	ADR: Abdominal pain, diarrhea, nausea, headache and respiratory tract infection	Patients with gastric ulcer (520)	Valenzuela et al., 1996^52^
	ADR: Diarrhea, headache, melena, chills and mononucleosis infection plus allergic reaction	Patients with duodenal ulcer (381)	Labenz et al., 1997^53^
	ADR: Stroke, cancer, pulmonary embolism and gastrointestinal bleeding/perforation	Patients with peptic ulcer with bleeding (274)	Muckadell et al., 1997^39^
	ADR: Cardiovascular events such as myocardial infarction, heart failure, stroke, pulmonary embolism, gastrointestinal bleeding and cancer	Patients with peptic ulcer in the stomach or duodenum (333)	Hasselgren et al., 1997^54^
	ADR: Diarrhea, stomatitis, metallic taste in the mouth and abdominal pain	Patients with active gastric or duodenal ulcer (78)	Annibale et al., 1997^55^
	ADR: Epigastric pain, facial erythema and loss of libido	Patients with erosive or ulcerative esophagitis, grade 2 or 3 (231)	Annibale et al., 1998^56^
	ADR: Dizziness, fatigue and aphthous stomatitis	Outpatients with symptoms of reflux esophagitis (70)	Ladas et al., 2000^57^
	TI: Omeprazole failed to control the gastric acidity of some patients	Patients with gastroesophageal reflux disease (88)	Leite et al., 1998^58^
	ADR: Diarrhea, taste disorder, increased levels of liver enzymes and cholecystitis	Patients diagnosed with at least one duodenal ulcer and with a test for *H. pylori* (539)	Lind et al., 1999^61^
	ADR: Death due to cardiovascular problems	Patients with persistent reflux esophagitis and who did not respond to treatment with H2 receptor antagonists (230)	Klinkenberg-Knol et al., 2000^60^
	ADR: Diarrhea, nausea, headache, cold, vomiting and fever	Patients with gastroesophageal reflux without erosive esophagitis (359)	Richter et al., 2000^59^
Clinical trials (28)	TI/ADR: Worsening of symptoms; taste disorder and scarlet fever	Patients with chronic functional dyspepsia with or without gastritis due to *H. pylori* (974)	Blum et al., 2000^62^
	ADR: Diarrhea, pericarditis and chest pain	Patients with erosive gastroesophageal reflux cured within 90 days (243)	Thjodleifsson et al., 2000^63^
	ADR: Diarrhea, abdominal pain and headache	Patients with dyspeptic symptoms (73)	Gottrand et al., 2001^33^
	TI: Some patients did not respond to treatment with omeprazole	Patients with dyspepsia (514)	Rabeneck et al., 2002^64^
	ADR: Increased fibroblast growth factor 2	Patients with gastric neoplasm (16)	Esaki et al., 2002^65^
	ADR: Myocardial infarction, ventral hernia, deep vein thrombosis, miscarriage, headache, respiratory infection, diarrhea and abdominal pain	Patients who suffered with burning in the stomach for at least three months (390)	Armstrong et al., 2005^66^
	ADR: Diarrhea, taste disorders and dyspepsia	Patients infected with *H. pylori* with abdominal disorders (323)	Manes et al., 2005^38^
	ADR: Nasopharyngitis, upper respiratory tract inflammation, diarrhea, headache, arthralgia, back pain, insomnia, cystitis, abdominal pain and hyperglycemia	Japanese patients with recurrent reflux esophagitis (119)	Ohkusa et al., 2005^67^
	ADR: Allergic reaction	Patients with lymphocytic gastritis (51)	Madisch et al., 2006^68^
	ADR/TI: Headache, somnolence and diarrhea	HIV-negative, healthy patients (19)	Schöller-Gyüre et al., 2008^32^
	ADR: Headache and gastrointestinal disorders	Patients with burning in the stomach or reflux (55)	Howden et al., 2009^69^
	ADR: Increased weight, increased ferritin level, increased death related to cardiac disorders and non-fatal heart attack	Patients with esophageal reflux (310)	Lundell et al., 2009^70^
	ADR: Omeprazole reduced antiplatelet effects	Unmedicated male patients (24)	Ferreiro et al., 2010^71^
	DI: Increased levels of leukocytes and platelets and increased incidence of cardiac and cerebral adverse events	Patients with stent implantation (38)	Hudzik et al., 2010^72^
	ADR: Diarrhea, tiredness, dizziness, abdominal pain and headache	Patients with typical symptoms of reflux more than twice a week (200)	Miwa et al., 2011^73^
Cohort studies (17)	ADR: Thrombosis, hyperthyroidism, complete retinal detachment, ulcerative colitis and skin rash	Patients with persistent reflux esophagitis and who did not respond to treatment with H_2_ receptor antagonists (178)	Klinkenberg-Knol et al., 1994^74^
	ADR: Death due to cardiovascular, cerebrovascular, respiratory and postoperative problems, carcinomas, urinary tract infections and suicide	Diagnosed with colitis due to *C. difficile* (140)	Cadle et al., 2007^13^
	ADR: Myocardial infarction, stroke, cardiovascular death and unstable angina	Patients using clopidogrel after percutaneous coronary intervention (16,690)	Kreutz et al., 2010^75^
	DI: Inhibition of the effect of clopidogrel	Patients using clopidogrel (18,139)	van Boxel et al., 2010^76^
	DI: Increased anticoagulant effect of acenocoumarol	Patients that used acenocoumarol for at least 42 days in the study period (2,755)	Teichert et al., 2011^77^
	TI: Cardiovascular death, myocardial infarction and stroke	Patients who underwent coronary intervention (13,144)	Kimura et al., 2011^78^
	ADR: Increased levels of chromogranin A	Patients with increased levels of chromogranin A that could not be caused by neuroendocrine tumors (196)	Korse et al., 2011^79^
	ADR: Hypergastrinemia	Patients with moderate to severe peptic esophagitis	Ligumsky et al., 2011^80^
	TI: Omeprazole failed to control the gastric acidity of some patients	Patients who underwent kidney transplantation	David-Neto et al., 2012^49^
	DI: Inhibition of the effect of clopidogrel	Patients with acute coronary syndrome (37,099)	Lin et al., 2012^81^
	ADR: Increased risk of fractures	Patients who underwent medical consultations in the last two years (61,916)	Soriano et al., 2014^16^
	ADR: Increased risk of dementia	Elderly people over 75 years old (73,679)	Gomm et al., 2016^44^
	ADR: Increased risk of first-time ischemic stroke	- (396,296)	Yi et al., 2017^47^
	ADR: Increased serum creatinine levels	Inpatient patients (419)	Varallo et al., 2018^41^
	ADR: Increased risk of metabolic syndrome and hepatic steatosis	Patients with a recent diagnosis of celiac disease (301)	Imperatore et al., 2018^45^
	ADR: Hypomagnesemia	Hospitalized patients with Torsades de pointes (48)	Lazzerini et al., 2018^42^
	ADR: Increased risk of fibrosis progression, cirrhosis, hepatic decompensation and development of hepatocellular carcinoma	Patients with hepatitis C virus (HCV) infection.	Li et al., 2018^48^
Quasi-experimental studies (4)	ADR: Diarrhea and ringing in the ears	Patients with burning in the stomach, erosive esophagitis or non-erosive reflux disease (108)	Tsuzuki et al., 2011^82^
	ADR: Respiratory infection, otitis media, pharyngitis, change in bowel habit, fever and rhinitis	Patients with cured reflux esophagitis (64)	Hassall et al., 2012^83^
	ADR: Nausea, vomiting, constipation, diarrhea, metallic taste in the mouth, headache, abdominal pain, loss of appetite, drowsiness, weakness, dizziness and dry mouth	Patients with *H. pylori* (134)	Sezgin et al., 2014^34^
	ADR: Myocardial infarction or heart failure with or without consequent death	Patients who were hospitalized due to myocardial infarction within 12 weeks after starting use of proton-pump inhibitors (5,550)	Juurlink et al., 2013^84^
Case-control studies (2)	ADR: Maculopapular rash, angioedema and/or urticaria, Steven-Johnson’s syndrome, erythema multiforme, eczematous eruption and urticarial vasculitis	Patients with dyspepsia, gastroesophageal reflux disease and upper gastrointestinal tract bleeding; prevention of ulcers induced by nonsteroidal anti-inflammatory drugs, stress and prednisolone (170)	Chularojanamontri et al., 2012^85^
	ADR: Low sperm motility	Men who were planning to have children (955)	Heijgen et al., 2016^46^
Cross-sectional studies (2)	ADR: Proliferative changes	Patients who underwent endoscopy and who had been using proton-pump inhibitors for at least 2 months (22)	Menegassi et al., 2010^5^
	ADR: Decreased serum levels of vitamin B12	Patients with diagnosis of gastrointestinal disease in the consumption of proton pump inhibitors (109)	Mindiola et al., 2017^40^
No clinical outcomes	Clinical trials (12)	Many conditions	Yamamoto et al., 1995^86^; Goh et al., 1995^87^; Soga et al., 1999^37^; Noordzij et al., 2001^88^; Zhou et al., 2002^43^; van Zanten et al., 2005^35^; Fujiwara et al., 2005^89^; Liu et al., 2013^90^; Miner JR et al., 2010^91^; Ummarino et al., 2012^36^; Sakurada et al., 2012^92^; Solana et al., 2013^93^
	Cohort studies (5)	Many conditions	Zairis et al., 2010^94^; Harjai et al., 2011^95^; Chen et al., 2014^28^; Galante et al., 2012^96^; Wang et al., 2017^97^
	Cross-sectional studies (2)	Newborns with hypospadias born to mothers who had used proton-pump inhibitors during pregnancy (430,569)	Erichsen et al., 2014^98^
		Patients with stage 5 chronic kidney disease (CKD) on hemodialysis therapy and chronic use of proton pump inhibitors (37)	Restrepo et al., 2017^99^

ADE = adverse drug event; ADR = adverse drug reaction; DI = drug interaction; TI = therapeutic ineffectiveness.

Among the 40 clinical trials included in the review, after risk-of-bias analysis, it was found that eight were classified as presenting low risk of bias, 14 as having high risk of bias and 17 as having uncertain risk of bias. The 17 studies analyzed using the Newcastle-Ottawa scale had low risk of bias (Table 3).

**Table 3.** Assessment of risk of bias in clinical trials using the RoB 1.0 tool and evaluation of quality of cohort and control case studies using the Newcastle-Ottawa scale

	Study	Risk of bias						
		Random sequence generation	Allocation concealment	Blinding of participants and personnel	Blinding of outcome assessors	Incomplete outcome data	Selective outcome reporting	Other sources of bias
Clinical trials (n = 39)	Yamamoto et al., 1995^86^	Unclear	High	High	High	Low	Low	Low
	Bate et al., 1995^50^	Unclear	Unclear	Low	Low	Low	Low	Low
	Goh et al., 1995^87^	Unclear	Unclear	Unclear	Low	Unclear	Low	Low
	Marzio et al., 1995^51^	Unclear	Unclear	Low	Low	Low	Low	Low
	Leite et al., 1996^58^	Unclear	Unclear	Unclear	Unclear	Low	Low	Low
	Valenzuela et al., 1996^52^	Unclear	Unclear	Low	Low	Low	Low	Low
	Muckadell et al., 1997^39^	Low	Low	Low	Low	Low	Low	Low
	Labenz et al., 1997^53^	Low	Unclear	Low	Low	Low	Low	Low
	Annibale et al., 1997^55^	Unclear	Unclear	Low	Low	Low	Low	Low
	Hasselgren et al., 1997^54^	Unclear	Low	Low	Low	Low	Low	Low
	Lind et al., 1999^61^	Unclear	High	Low	Low	Low	Low	Low
	Soga et al., 1999^37^	Low	Low	Unclear	Unclear	Low	Low	Low
	Klinkenberg-Knol et al., 2000^60^	High	Unclear	Unclear	Low	High	Low	Low
	Ladas et al., 2000^57^	Low	High	Low	High	Low	Low	Low
	Richter et al., 2000^59^	Unclear	Unclear	Low	Unclear	Low	Low	Low
	Blum et al., 2000^62^	Unclear	Low	Low	Low	Low	Low	Low
	Noordzij et al., 2011^88^	Unclear	Low	Low	Low	Low	Low	Low
	Gottrand et al., 2001^33^	Low	Low	Low	Low	Low	Low	Low
	Esaki et al., 2002^65^	Unclear	Unclear	Low	Low	Low	Low	Low
	Rabeneck et al., 2002^64^	Low	Low	Low	Low	Low	Low	Low
	Zhou et al., 2002^43^	Unclear	Unclear	High	High	Low	Low	Low
	Thjodleifsson et al., 2000^63^	Low	Low	Low	Low	Low	Low	Low
	Armstrong et al., 2005^66^	Low	Low	Low	Low	Low	Low	Low
	Fujiwara et al., 2005^89^	Unclear	Unclear	High	High	Low	Low	Low
	Manes et al., 2005^38^	Low	Unclear	Low	High	Low	Low	Low
	Ohkusa et al., 2005^67^	High	High	Unclear	Unclear	Low	Low	Unclear
	Van Zanten et al., 2005^35^	Low	Low	Low	Low	Low	Low	Low
	Madisch et al., 2005^68^	Low	Low	Low	Low	Low	Low	Low
	Schooler et al., 2008^32^	Low	Unclear	Low	High	Low	Low	Low
	Howden et al., 2009^69^	Unclear	Unclear	Unclear	High	Low	Low	Low
	Lundell et al., 2009^70^	Unclear	Unclear	Low	Unclear	Low	Low	Low
	Miner et al., 2010^91^	Low	Low	Low	Low	Low	Low	Low
	Hudzik et al., 2010^72^	High	High	Low	High	Low	Low	Low
	Ferreiro et al., 2010^71^	Unclear	Unclear	Low	Low	Low	Low	Low
	Miwa et al., 2011^73^	Low	Low	Low	Low	Low	Low	Low
	Sakurada et al., 2012^92^	Unclear	Low	Low	High	Low	Low	Low
	Ummarino et al., 2011^36^	Unclear	Unclear	Unclear	Unclear	Low	Low	Low
	Liu et al., 2013^90^	Low	Unclear	Low	High	Low	Low	Low
	Solana et al., 2013^93^	Low	Unclear	Unclear	Unclear	Low	Low	Low
	Evaluation of quality of cohort and control case studies using the Newcastle-Ottawa scale							
	Study		Domains					
			Selection (4*)		Comparability (2*)		Outcome (3*)	
Cohort studies (22)	Klinkenberg-Knol et al., 1994^74^	4*			1*		3*	
	Kreutz et al., 2010^75^	4*			2*		3*	
	Van Boxel et al., 2010^76^	4*			2*		3*	
	Zairis et al., 2010^94^	4*			2*		3*	
	Cadle et al., 2007^13^	4*			2*		3*	
	Study		Domains					
			Selection (4*)		Comparability (2*)		Outcome (3*)	
Cohort studies (22)	Teichert et al., 2011^77^	4*			2*		3*	
	Ligumsky et al., 2011^80^	3*			2*		3*	
	Galante et al., 2012^96^	4*			1*		3*	
	Lin et al., 2012^81^	4*			2*		3*	
	David-Neto et al., 2012^49^	4*			1*		3*	
	Soriano et al., 2014^16^	4*			2*		3*	
	Chen et al., 2014^28^	4*			2*		3*	
	Wang et al., 2017^97^	4*			2*		3*	
	Gomm et al., 2016^44^	4*			2*		3*	
	Yi et al., 2017^47^	4*			2*		3*	
	Varallo et al., 2018^41^	4*			2*		3*	
	Imperatore et al., 2018^45^	4*			2*		3*	
	Lazzerini et al., 2018^42^	4*			1*		3*	
	Li et al., 2018^48^	4*			2*		3*	
	Study		Domains					
			Selection (4)		Comparability (2*)		Exposure (3*)	
Case-control study (2)	Chularojanamontri et al., 2012^85^	4*			2*		3*	
	Heijgen et al., 2016^46^	4*			2*		3*	

## DISCUSSION

This review allowed us to identify and update the most severe and prevalent ADEs relating to use of omeprazole, and our findings corroborate similar results found in other studies.^3^^,^^4^ Severe ADEs occurred in patients who underwent heart-related surgery or drug interventions, such as in situations of acute coronary syndromes or percutaneous coronary intervention,^75^^,^^78^^,^^96^ or in cases of concomitant use of such medications.^76^ These events were associated with concomitant use of omeprazole and clopidogrel, which caused inhibition of the antiplatelet effect of omeprazole,^83^ due to competitive inhibition of CYP2C19.^32^

Several drug interactions relating to omeprazole, especially with antiplatelet agents, are known.^78^^,^^94^ The non-serious events that have been described are diarrhea, headache and somnolence, relating to use of omeprazole concomitantly with the antiretroviral drug etravirine.^32^ The severe adverse events that have been described comprise inhibition of the antiplatelet effects of drugs such as clopidogrel, which increases the risk of developing heart problems that may lead to death; and decreased absorption of mycophenolic acid, which leads to rejection of transplanted organs.^49^

Nevertheless, it is not possible to say with certainty that the adverse events described in these studies occurred due to drug interactions with omeprazole, since some of the studies included did not present statistically significant results.^71^^,^^94^^,^^95^^,^^96^

In two studies in which omeprazole was added to dual antiplatelet therapy (a combination of clopidogrel and acetylsalicylic acid), it reduced the stomach pain resulting from this therapy and no risk was found in this combination.^95^ Nonetheless, it is always necessary to monitor potentially dangerous drug combinations between omeprazole and clopidogrel, acetylsalicylic acid and mycophenolate mofetil, among others.

Regarding drug interactions, all patients may be exposed to their effects, regardless of age or clinical condition. However, some patients are more susceptible, such as those who already have some type of heart disease or the elderly, who commonly use polypharmacy.

Only 12 studies included elderly patients, and these studies reported occurrences of severe adverse events such as dementia, myocardial infarction, cardiovascular death, stroke and pulmonary embolism, among others. In the non-elderly population, the severe adverse events reported included myocardial infarction, stroke, death and pulmonary embolism, but no relationship between the severity or the frequency of events and the patients’ age was observed from use of omeprazole. However, other authors have suggested that age is a factor that influences occurrences of adverse events. Varallo et al.^24^ observed in a cross-sectional study that the elderly population had fewer ADEs than adults did, probably because doctors provide greater care and attention regarding pharmacotherapeutic management for patients of this age group, since there are other factors that increase the likelihood of ADEs, such as polypharmacy. Beijer and de Blaey^100^ reported that the chances that elderly individuals would need to be hospitalized due to adverse drug reactions (ADRs) were four times higher than those of younger people (16.6% versus 4.1%). Additionally, in 2015, the American Geriatrics Society advised through the Beers criteria that unjustified use of PPIs among the elderly for more than eight weeks should be avoided, since exposure to such drugs increases the risks of infection by *Clostridium difficile*, bone loss and fractures.^13^^,^^16^^,^^101^

Another factor that may have influenced the appearance of adverse events is the duration of use of omeprazole. Non-serious adverse events such as diarrhea, headache, flatulence and abdominal pain, among others, have been reported among patients taking omeprazole for short periods of time, i.e. from a few days of use to a maximum of two weeks.^32^^,^^33^^,^^34^^,^^35^^,^^36^^,^^74^ Severe adverse events have been reported among patients who used omeprazole for longer times, i.e. more than one month.^35^^,^^36^^,^^37^^,^^54^^,^^57^^,^^60^^,^^63^^,^^65^^,^^66^^,^^67^^,^^70^^,^^71^^,^^74^^,^^75^^,^^76^^,^^70^^,^^95^^,^

In only one of the studies analyzed here was omeprazole prescribed for off-label use.^41^ However, off-label prescription of omeprazole is widespread in many countries and there is a need to assess the safety of this use. We take the view that the duration of exposure is likely to increase the likelihood of adverse events, since polypharmacy alone is a risk factor for occurrences of adverse events.^24^

Outcomes of therapeutic ineffectiveness and symptom worsening were identified. It was noted that some patients did not respond to omeprazole treatment^13^^,^^32^^,^^64^^,^^70^ and that for others, their symptoms worsened.^62^ The most likely reason for such events would be high concentrations of acid in the stomach, which could cause gastroparesis, decrease absorption and, consequently, decrease the therapeutic effect of omeprazole.

Although most of the adverse events reported were already known, unexpected events such as dementia,^44^ low-motility sperm,^46^ miscarriage, proliferative changes,^5^ increased levels of chromogranin A,^79^ increased levels of fibroblast growth factor 2,^72^ chills, cardiovascular events (myocardial infarction, heart failure, stroke, ischemic stroke, pulmonary embolism and thrombosis),^47^ scarlet fever, hyperglycemia, mononucleosis infection, gastrointestinal bleeding, nasopharyngitis, otitis media, loss of libido and rhinitis have also been identified.^4^^,^^65^^,^^66^^,^^67^^,^^102^ Because the associations between these adverse events and use of omeprazole are not fully understood, there is a need to carry out further studies to investigate the relationships between omeprazole and these events. If such associations are verified, they should be described in the package leaflet.

In addition, more recent studies have identified other adverse events, such as decreased vitamin B12 levels,^40^ increased levels of creatinine^41^ and hypomagnesia.^42^

Use of omeprazole is considered safe in the following situations: when it is not combined with antiplatelet drugs; when it is administered to replace H2 receptor antagonists in patients who are resistant to treatment with drugs of this class; when the most appropriate posology and dosage is established for each condition and patient; and when omeprazole is used in conjunction with a combination of antibiotics to eradicate *H. pylori* and to treat esophagitis, among other situations.^94^^,^^102^

### Limitations of the present study

No *a priori* design was provided for this review and the languages were restricted to Portuguese, English and Spanish.

Gray literature was not included. However, its inclusion would be unviable and probably would not add to the results found, since this type of literature is characterized by incomplete and poorly constructed data.

No methods were used to assess the homogeneity or heterogeneity between the studies, and the risk of publication bias among the studies included was not assessed. Furthermore, no information regarding potential conflicts of interest in the primary studies included was available.

All the outcomes evaluated related to approved indications for use of omeprazole. Therefore, the data confirm that there is no evidence of clinical outcomes (safety and effectiveness) resulting from unapproved use of omeprazole, such as polypharmacy (although polypharmacy is commonly used). The duration of use of omeprazole influenced occurrences of adverse events. Severe adverse events, such as death, stroke and myocardial infarctions occurred during prolonged treatments (more than one month). Non-serious adverse events occurred over short periods (from a few days to a maximum of two weeks). Use of omeprazole needs to be monitored primarily in patients with heart disorders who are using antiplatelet agents and omeprazole concomitantly and in newly transplanted patients who are using mycophenolic acid as a suppressive agent, in order to avoid severe adverse reactions such as organ transplant rejection, death, stroke and myocardial infarction.

## CONCLUSION

Therefore, use of omeprazole can be considered safe in the following situations: when it is not combined with antiplatelet drugs; when it is administered to replace H2 receptor antagonists in patients who are resistant to treatment with drugs of this class; when the posology is well established for each condition and type of patient; and when omeprazole is used to eradicate *H. pylori*, among others. Most of the trials included in this review presented uncertain risk or high risk of bias, which indicates that there is a need for better-designed studies. The high risk of bias related mainly to the blinding of the participants and outcome assessors. It should be noted that if patients and professionals believe that omeprazole is a gastric protector and is risk-free, this may lead to bias in the analysis and to under identification and underreporting of adverse events relating to omeprazole. This may suggest that the existing studies may have underestimated the adverse events.
